# Supplementary material for: Met-ID: An Open-Source Software for Comprehensive Annotation of Multiple On-Tissue Chemical Modifications in MALDI-MSI
Source: Anal Chem. 2025 Apr 20;97(16):9033–41. doi: 10.1021/acs.analchem.5c00633 (PMC12044586; doi:10.1021/acs.analchem.5c00633)
Supplement: Supplementary file 1 — ac5c00633_si_001.pdf [file ac5c00633_si_001.pdf]

SUPPORTING INFORMATION

Met-ID: An Open-Source Software for Comprehensive Annotation of Multiple On-Tissue Chemical Modifications in MALDI-MSI

Patrik Bjärterot[1], Anna Nilsson[1], Reza Shariatgorji[1], Theodosia Vallianatou[1], Ibrahim Kaya[1], Per Svenningsson[2], Lukas Käll[3], and Per E. Andrén[1*]

[1] Dept. of Pharmaceutical Biosciences, Spatial Mass Spectrometry, Science for Life Laboratory, Uppsala University, SE-75124 Uppsala, Sweden, [2] Dept. of Clinical Neuroscience, Karolinska Institute, SE-17177 Stockholm, Sweden, and [3]Science for Life Laboratory, School of Engineering Sciences in Chemistry, Biotechnology and Health, Royal Institute of Technology-KTH, SE-17165 Solna, Sweden.

[*]Corresponding author. Email: per.andren@uu.se

SUPPORTING FIGURES		Page S2-S6
Figure S1	Isomers of dopamine.	Page S2
Figure S2	Derivatives of FMP-10.	Page S2
Figure S3	Product ions specific to FMP-10 derivatization of different functional groups.	Page S3
Figure S4	Mirrored MS2 spectra comparing tissue metabolites to Met-ID database spectra.	Page S4-S6

SUPPORTING TABLES		Page S7-S9
Table S1	List of FMP-10 chemical standards and number of fragments.	Page S7
Table S2	List of FMP-10 *m*/*z* ratios used in calibration of an MS1 experiment on chemical standards.	Page S7
Table S3	List of FMP-10 *m*/*z* ratios used in calibration of an MS1 experiment on tissue.	Page S8
Table S4	Comparison of delta ppm error between FMP-10 searches on tissue data.	Page S8
Table S5	List of negative mode *m*/*z* ratios used in calibration of an MS1 experiment.	Page S8
Table S6	Comparison of tissue-derived MS2 spectra to MS2 spectra of standards in the Met-ID database with the maximum *m/z* ratio limit.	Page S9
Table S7	Comparison of tissue-derived MS2 spectra to MS2 spectra of standards in the Met-ID database using different bin sizes.	Page S9

SUPPORTING METHODS		Page S10
Method S1	HTX sprayer methods.	Page S10
Method S2	MALDI-MSI of rat brain tissue with FMP-10.	Page S10
Method S3	MALDI-MSI of rat brain tissue in negative mode.	Page S10
Method S4	MALDI-MSI of chemical standards with FMP-10	Page S10

Supporting Figures

421.1911 | Show only identified | Search...

	Observed m/z	Adjusted m/z	Matched name	Adduct	Formula	Matched delta mass (Da)	Matched difference (ppm)	Matched theoretical mass	MSMS available?	Coverage
	421.191100	421.191100	2-(2-Amino-1-hydroxyethyl)phenol	[M+FMP10]	C8H11NO2	4.54020e-5	0.107794	421.191055	Not available	1 / 3
	421.191100	421.191100	Norfenefrine	[M+FMP10]	C8H11NO2	4.54020e-5	0.107794	421.191055	Not available	1 / 3
	421.191100	421.191100	Dopamine	[M+FMP10]	C8H11NO2	4.53990e-5	0.107787	421.191055	Available	1 / 6
	421.191100	421.191100	p-Octopamine	[M+FMP10]	C8H11NO2	4.53990e-5	0.107787	421.191055	Available	1 / 3
	421.191100	421.191100	Vanillylamine	[M+FMP10]	C8H11NO2	4.53990e-5	0.107787	421.191055	Not available	1 / 3

Figure S1. Isomers of dopamine. Met-ID shows all isomers of dopamine that may be derivatized by FMP-10. In this example, the *m/z* value of single derivatized dopamine was queried. Met-ID displayed all the isomers, as well as which metabolites have MS2 spectra collected in the database.

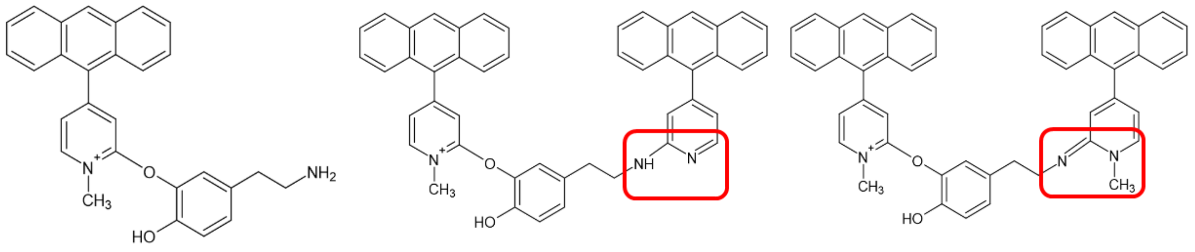

(a) Dopamine 1A *m/z* 421.1911

(b) Dopamine 2A *m/z* 674.2802

(c) Dopamine 2B *m/z* 688.2959

Figure S2. Derivatives of FMP-10. (a) Single-derivatized dopamine (dopamine 1A), and (b) double-derivatized dopamine (dopamine 2A). As highlighted by the red box, the FMP-10 molecule in dopamine 2A loses a methyl group, resulting in the loss of one positive charge and giving the molecule an overall charge of +1. (c) Double-derivatized dopamine (Dopamine 2B) retains the methyl group but loses a hydrogen. This causes a double bond to shift from the aromatic ring to the dopamine nitrogen. Consequently, the FMP-10 nitrogen loses its positive charge, resulting in an overall charge of +1. Both A and B derivatives can form if the target molecule undergoes double derivatization. Triple derivatization follows a similar pattern, with the addition of an additional derivative; C (e.g., dopamine 3A, dopamine 3B and dopamine 3C). The letters assigned to these derivatives correspond to their *m/z* ratios, with A representing the lowest *m/z* ratio and B and C representing progressively higher *m/z* ratios for double- and triple-derivatized molecules, respectively.

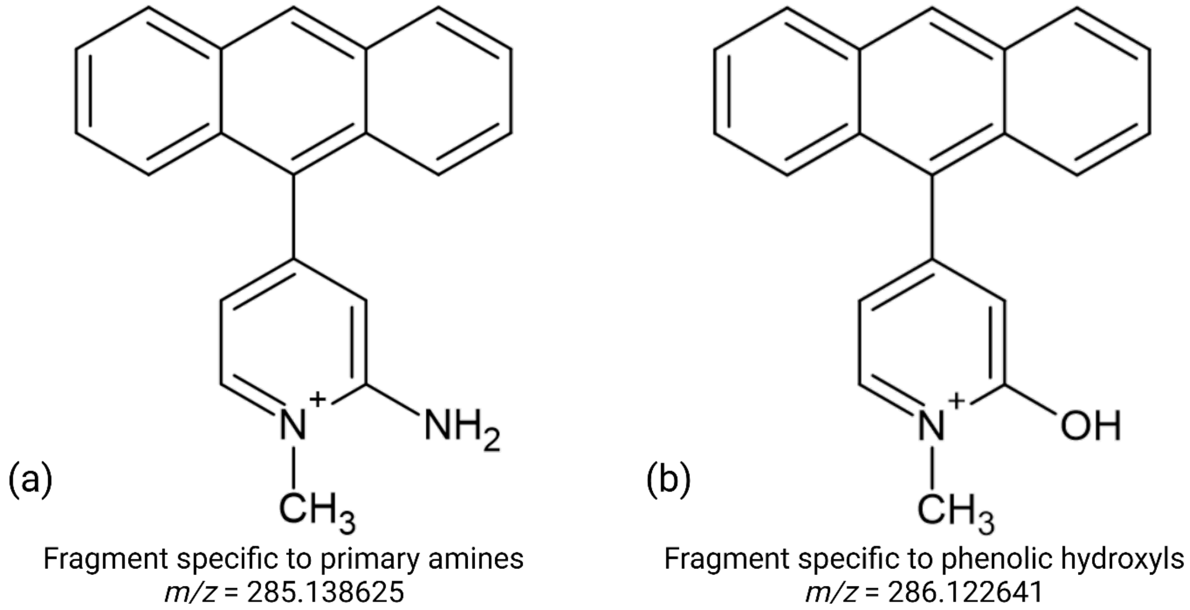

Figure S3. Product ions specific to FMP-10 derivatization of different functional groups. (a) Fragment specific to primary amine derivatization. (b) Fragment specific to phenolic hydroxyl derivatization.

a

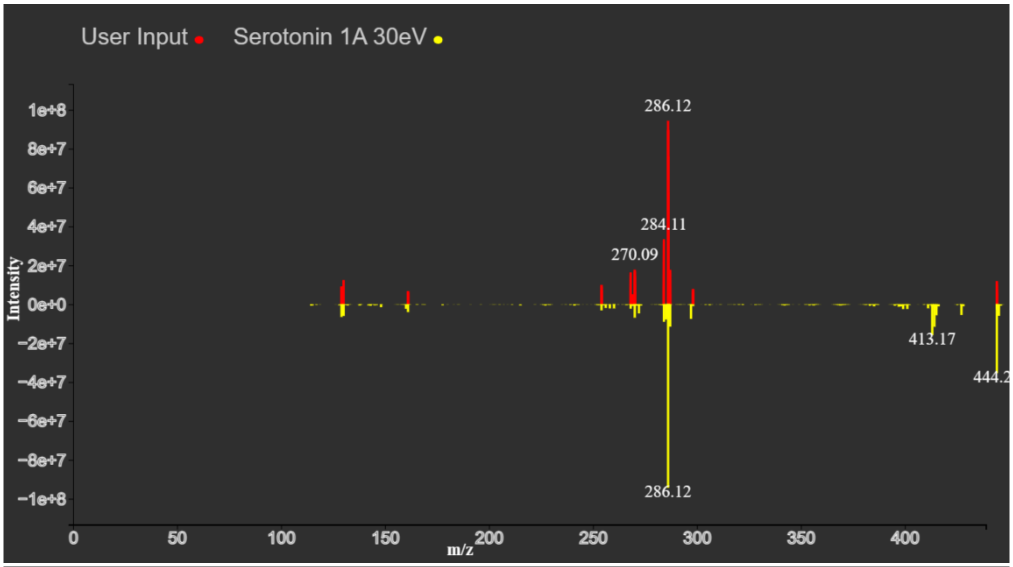
User Input
Serotonin 1A 30eV
Intensity
m/z
286.12
284.11
270.09
413.17
444.2
286.12
1e+8
8e+7
6e+7
4e+7
2e+7
0e+0
−2e+7
−4e+7
−6e+7
−8e+7
−1e+8
0
50
100
150
200
250
300
350
400

b

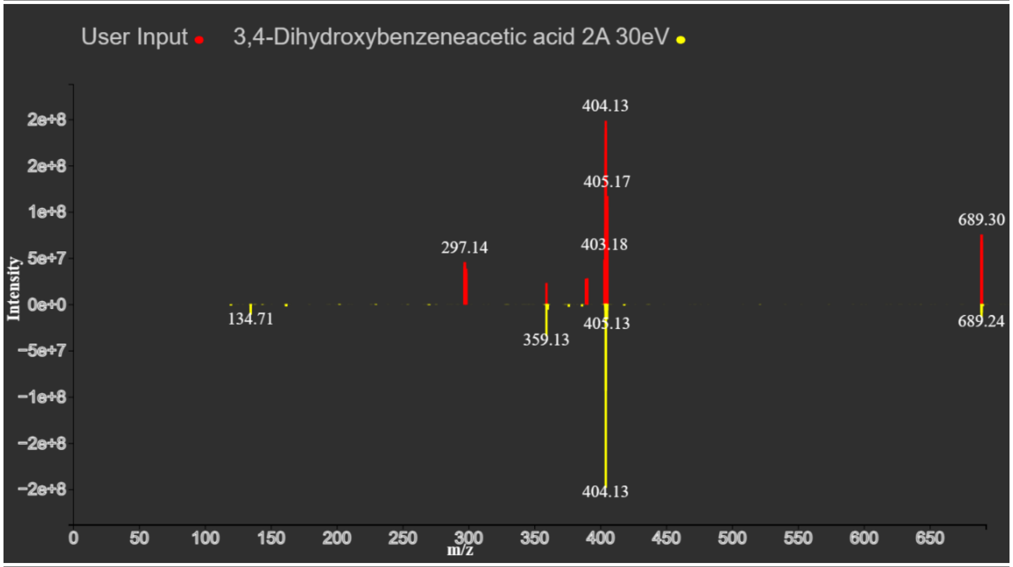
User Input
3,4-Dihydroxybenzeneacetic acid 2A 30eV
Intensity
m/z
404.13
405.17
403.18
297.14
689.30
134.71
359.13
405.13
689.24
404.13
2e+8
2e+8
1e+8
5e+7
0e+0
−5e+7
−1e+8
−2e+8
−2e+8
0
50
100
150
200
250
300
350
400
450
500
550
600
650

c

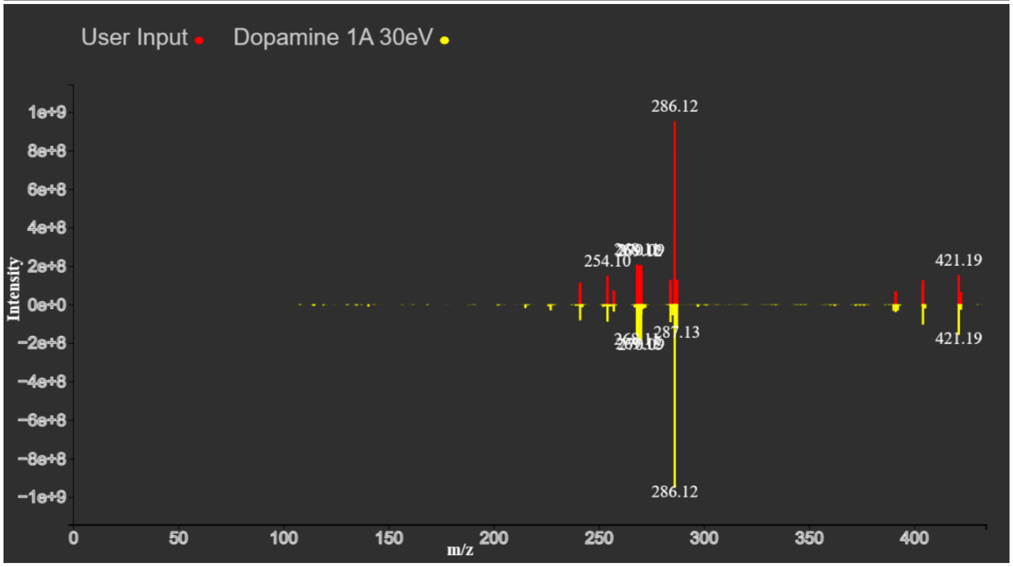
User Input
Dopamine 1A 30eV
Intensity
m/z
286.12
254.10
421.19
287.13
421.19
286.12
1e+9
8e+8
6e+8
4e+8
2e+8
0e+0
−2e+8
−4e+8
−6e+8
−8e+8
−1e+9
0
50
100
150
200
250
300
350
400

d

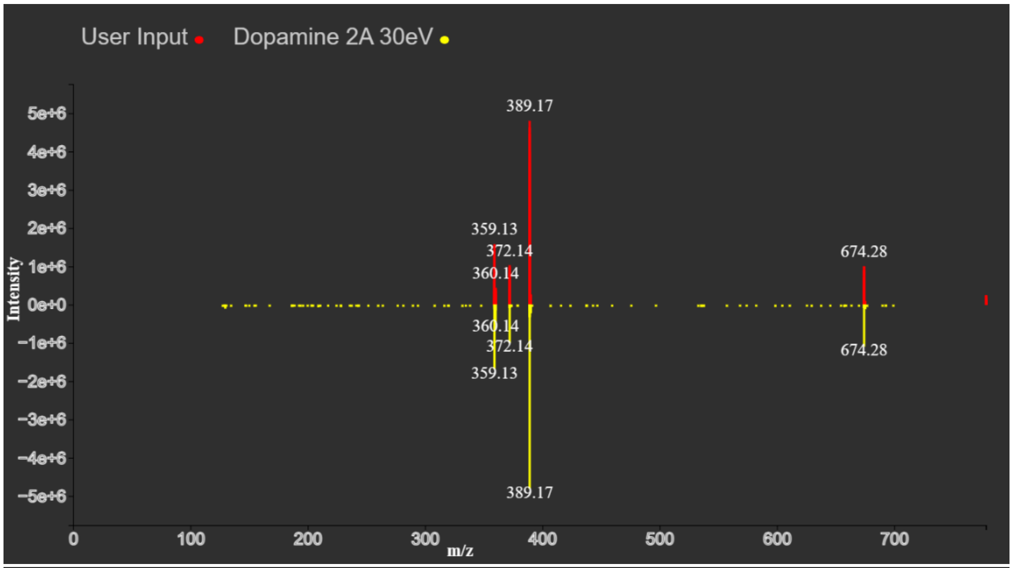

User Input
Dopamine 2A 30eV
Intensity
m/z
389.17
359.13
372.14
360.14
674.28

e

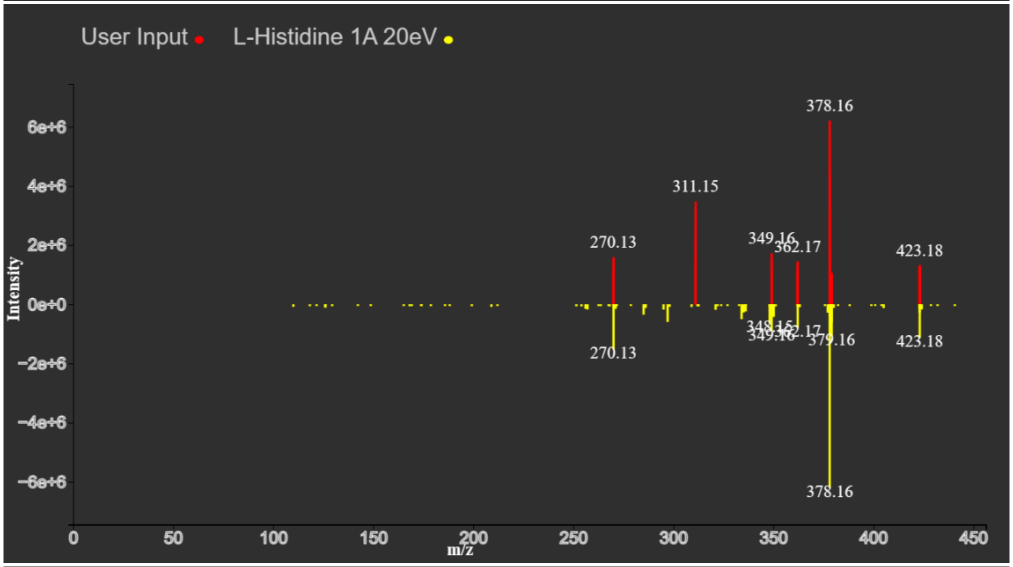

User Input
L-Histidine 1A 20eV
Intensity
m/z
378.16
311.15
270.13
349.16
362.17
423.18
379.16

f

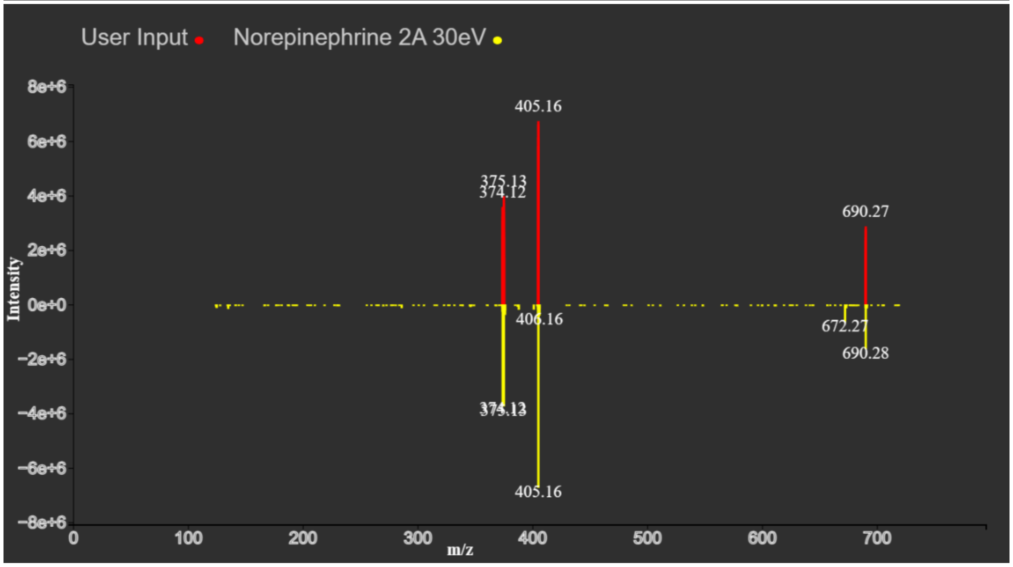

User Input
Norepinephrine 2A 30eV
Intensity
m/z
405.16
375.13
374.12
690.27
406.16
672.27
690.28

g

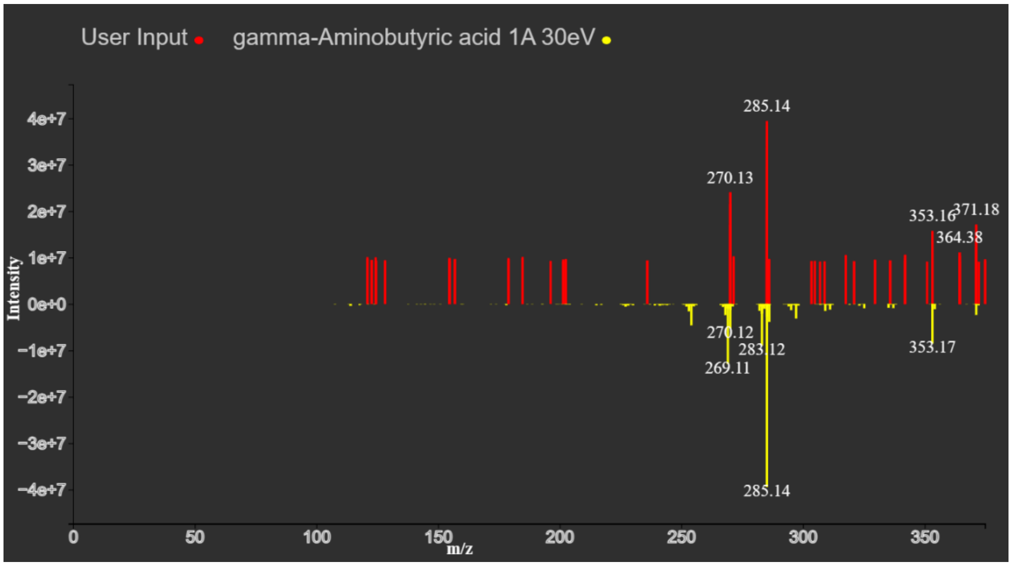

Figure S4. Mirrored MS2 spectra comparing tissue metabolites to Met-ID database spectra. The mirrored spectra are as follows (a) serotonin 1A 30 eV, (b) DOPAC 2A 30 eV, (c) dopamine 1A 30 eV, (d) dopamine 2A 30 eV, (e) histidine 1A 20 eV, (f) norepinephrine 2A 30 eV, and (g) GABA 1A 30 eV. The labeling of derivatives is explained in the caption of Figure S2.

Supporting Tables

Table S1. List of FMP-10 chemical standards and number of fragments. Certain chemical standards fragment more frequently, occasionally producing fragments with *m/z* values similar to other metabolites or their fragments. Such fragments may appear as peaks in *m/z* feature lists, potentially resulting in false-positive identifications. For instance, homovanillic acid contributed 10 of the 170 peaks in the m/z feature list but was correctly identified only once (as homovanillic acid 1A). The names of the chemical standards are consistent with the metabolite names in the Human Metabolome Database (HMDB).

Chemical standard	Number of fragments	Chemical standard	Number of fragments
5-Hydroxyindoleacetic acid	17	Norepinephrine	4
3,4-Dihydroxyphenylglycol	14	Vanillylmandelic acid	4
Homovanillic acid	10	Metyrosine	3
Vanillactic acid	10	Histamine	3
L-Lysine	9	Adenine	3
L-Histidine	9	L-Tyrosine	3
L-Arginine	9	L-Dopa	3
Epinephrine	9	Normetanephrine	2
Serotonin	9	*p*-Octopamine	2
3,4-Dihydroxyphenylacetic acid (DOPAC)	8	Putrescine	2
Norsalsolinol	7	Spermidine	2
3-Methoxytyrosine	7	5,6-Dihydroxyindole	2
3-Methoxytyramine	7	Melevodopa	1
Carbidopa	7	γ-Aminobutyric acid	1
Salsolinol	6	Spermine	1
Dopamine	5	Tryptamine	1
Hordenine	5	4-Hydroxy-benzaldehyde	1
Tyramine	4	L-Phenylalanine	0

Table S2. List of FMP-10 *m/z* ratios used in calibration of an MS1 experiment on chemical standards. A curve was fitted to this data and used to recalibrate the input data to more closely match the theoretical *m*/z ratio.

Metabolite	Derivative (*)	Experimental *m/z*	Theoretical *m/z*	ppm difference
Dopamine	1A	421.19144	421.191055	0.91
Dopamine	2A	674.27992	674.280203	-0.42
Dopamine	2B	688.29543	688.295854	-0.62
Dopamine	3B	941.38171	941.385004	-3.5
Lock mass		555.22310	555.223104	0
GABA-H2O	1A	353.16516	353.164840	0.91
GABA	1A	371.17562	371.175405	0.58

(*) Derivative labels are as described in the caption of Figure S2.

Table S3. List of FMP-10 *m/z* ratios used in calibration of an MS1 experiment on tissue. A curve was fitted to this data and used to recalibrate the input data to more closely match the theoretical *m/z*.

Metabolite	Derivative (*)	Experimental *m/z*	Theoretical *m/z*	ppm difference
Dopamine	1A	421.1910	421.191055	-0.13
Dopamine	2A	674.2802	674.280203	0
Dopamine	2B	688.2954	688.295854	-0.66
Dopamine	3B	941.3851	941.385004	0.10
Lock mass		555.2231	555.22310	0
GABA-H2O	1A	353.1648	353.16484	-0.11
GABA	1A	371.1753	371.175405	-0.28

(*) Derivative labels are as described in the caption of Figure S2.

Table S4. Comparison of delta ppm error between FMP-10 searches on tissue data. The ppm difference between the theoretical and observed *m/z* for all *m/z* features with annotations for the four different searches. The *m/z* features used in the calibration were excluded.

	Endogenous metabolites (Δppm)	All metabolites (Δppm)
No mass error calibration	0.524	0.519
Mass error calibration	0.427	0.435
Reduction (%)	18.39	16.21

Table S5. List of negative mode *m/z* ratios used in calibration of an MS1 experiment. A curve was fitted to this data and used to recalibrate the input data to more closely match the theoretical *m/z*.

Metabolite	Adduct	Experimental *m/z*	Theoretical *m/z*	ppm difference
Aspartic acid	$[M-H]^-$	132.03033	132.030231	0.75
Glucose	$[M+Cl]^-$	215.03293	215.032789	0.66
Glutamine	$[M-H]^-$	145.06182	145.061866	-0.32
Glutathione	$[M-H]^-$	306.07669	306.07653	0.52
Taurine	$[M-H]^-$	124.00746	124.007388	0.58
LysoPE(22:6)	$[M-H]^-$	524.27837	524.278263	0.20
PE(38:4)	$[M-H]^-$	766.53898	766.539229	-0.32
PE(40:6)	$[M-H]^-$	790.53881	790.539229	-0.53
PI(38:4)	$[M-H]^-$	885.55043	885.549859	0.64
Ascorbic acid	$[M-H]^-$	175.02491	175.024812	0.56

Table S6. Comparison of tissue-derived MS2 spectra to MS2 spectra of standards in the Met-ID database with the maximum m/z ratio limit. This table is similar to Table 1 with a fixed maximum *m*/*z* ratio considered. Some MS2 spectra have collected data greater than the precursor ion mass, which impacts the cosine similarity. Notably, DOPAC showed a considerable increase in cosine similarity when disallowing higher *m*/*z* values from being considered.

Tissue metabolite	Derivative (*)	CID (eV)	Isolation window (Da)	Max *m*/*z*	Bin size (Da)	Best DB hit	Rank of correct hit	Cosine similarity
Serotonin	1A	30	1	445	0.01	Serotonin 1A 30 eV	1st	0.965
DOPAC	2A	30	1	690	0.01	DOPAC 2A 30 eV	1st	0.798
Dopamine	1A	30	1	422	0.01	Dopamine 1A 30 eV	1st	0.99
Histidine	1A	30	1	424	0.01	Histidine 1A 30 eV	1st	0.738
Norepinephrine	2A	30	1	691	0.01	Norepinephrine 2A 30 eV	1st	0.87
Taurine	1A	20	1	394	0.01	Taurine 1A 20 eV	1st	0.127
α-tocopherol	1A	45	1	699	0.01	α-tocopherol 1A 40 eV	1st	0.320

(*) Derivative labels are as described in the caption of Figure S2.

Table S7. Comparison of tissue-derived MS2 spectra to MS2 spectra of standards in the Met-ID database using different bin sizes. Dopamine and norepinephrine were compared to illustrate the impact of bin size variation. Larger bin sizes produced similar cosine similarity scores but reduced specificity. Conversely, smaller bin sizes lowered sensitivity and cosine similarity due to minor calibration variations, which can occur when spectra are acquired at different times.

Tissue metabolite	Derivative (*)	CID (eV)	Isolation window (Da)	Max *m*/*z*	Bin size (Da)	Best DB hit	Rank of correct hit	Cosine similarity of top hit & correct hit
Dopamine	1A	30	1	422	1	Dopamine 1A 30 eV	1st	0.989
Dopamine	1A	30	1	422	0.1	Dopamine 1A 30 eV	1st	0.989
Dopamine	1A	30	1	422	0.01	Dopamine 1A 30 eV	1st	0.99
Dopamine	1A	30	1	422	0.001	Dopamine 1A 30 eV	1st	0.794
Dopamine	1A	30	1	422	0.0001	DHPME 1A 30 eV	>20th	0.405&0.049
Norepinephrine	2A	30	1	691	1	Norepinephrine 2A 30 eV	1st	0.983
Norepinephrine	2A	30	1	691	0.1	Norepinephrine 2A 30 eV	1st	0.982
Norepinephrine	2A	30	1	691	0.01	Norepinephrine 2A 30 eV	1st	0.87
Norepinephrine	2A	30	1	691	0.001	Norepinephrine 2A 30 eV	1st	0.642
Norepinephrine	2A	30	1	691	0.0001	Epinephrine 2B 40 eV	5th	0.168&0.072

(*) Derivative labels are as described in the caption of Figure S2.

Supporting Methods

Method S1. HTX sprayer methods

N-(1-naphthyl) ethylenediamine dihydrochloride (NEDC) was prepared at a concentration of 7 mg/ml in 70 % MeOH and sonicated in an ultrasonic bath for 30 s. The matrix solvent was sprayed over the sample and the dried standard spots using a TM-Sprayer (HTX Technologies) in 14 passes with a 0.07 ml/min flow of pushing solvent (50% ACN), nozzle temperature of 50°C, track spacing of 2 mm, nozzle velocity of 1100 mm/min and N_2 gas pressure of 6 psi. The derivatizing matrix FMP-10 was used according to a previously described protocol [1,2]. In brief, 20 passes of 4.4 mM FMP-10 in 70% acetonitrile were sprayed (nitrogen pressure of 6 psi) in horizontal lines over the tissue with a solvent flow rate of 80 µL/min, nozzle temperature of 90 °C, nozzle velocity of 1100 mm/min and track spacing of 2 mm.

Method S2. MALDI-MSI of rat brain tissue with FMP-10

Mass spectrometry imaging data of rat brain tissue sections coated with FMP-10 were acquired at 50 µm lateral resolution on a timsTOF flex MALDI-2 mass spectrometer (Bruker Daltonics, Bremen, Germany). A single focus beam was used to collect 450 shots/pixel at 10 kHz. The scan range was set to 300 – 1000 *m*/*z* with TIMS on with the $1/K_0$ range set to 0.7-1.65 and a ramp time of 300 ms. The method was externally calibrated using red phosphorous and internally calibrated using the matrix peak at *m*/*z* 569.2224. Data was imported into SCiLS (Bruker Daltonics) and the T-ReX^2 peak-picking algorithm was used with 90% coverage and no noise filtering.

Method S3. MALDI-MSI of rat brain tissue in negative mode

A MALDI-FTICR-MS instrument (Solarix XR 7T-2Ω, Bruker Daltonics) was used for acquiring data from the NEDC-coated sections. The mass range was set to 86-1000 *m*/*z* and data was acquired at 100 µm lateral resolution, summing up 100 shots/pixel. The medium laser focus was used at a frequency of 1 kHz. The quadrupole isolation (Q1) *m*/*z* was set to 120 and the transfer optics and time-of-flight (TOF) was set to 4 MHz and 0.650, respectively. No lock mass was used. The method was externally calibrated using red phosphorous. The sqlite data format was imported into SCiLS and the T-ReX^2 feature-finding algorithm with no spatial noise filtering and 95% coverage was used to find features.

Method S4. MALDI-MSI of chemical standards with FMP-10

The same MALDI-FTICR-MS instrument was used for acquiring data from the chemical standards coated with FMP-10. The scan range was set to 150.50-1050 *m*/*z* data was collected at 200 µm lateral resolution summing up100 shots per pixel. The medium laser focus was used at a frequency of 1 kHz. The quadrupole isolation (Q1) *m*/*z* was set to 379 and the transfer optics and TOF were set to 4 MHz and 0.750 ms, respectively. The FMP-10 derived peak at *m*/*z* 555.2231 was used as lock mass for internal *m*/*z* calibration. The sqlite data format was imported into SCiLS and feature finding was performed using the T-ReX^2 feature finding algorithm with 90% coverage and weak noise filtering settings.

References

(1) Shariatgorji M, Nilsson A, Fridjonsdottir E, Vallianatou T, Källback P, Katan L, Sävmarker J, Mantas I, Zhang X, Bezard E, Svenningsson P, Odell LR, Andrén PE. Comprehensive mapping of neurotransmitter networks by MALDI-MS imaging. Nat. Methods **2019,** *16* (10), 1021-1028.

(2) Shariatgorji R, Nilsson A, Fridjonsdottir E, Strittmatter N, Dannhorn A, Svenningsson P, Goodwin RJA, Odell LR, Andrén PE. Spatial visualization of comprehensive brain neurotransmitter systems and neuroactive substances by selective in situ chemical derivatization mass spectrometry imaging. Nat. Protoc **2021**, *16* (7), 3298-3321.
